# Effects of Adjunct Questions on L2 Reading Comprehension with Texts of Different Types

**DOI:** 10.3390/bs14020138

**Published:** 2024-02-15

**Authors:** Yunmei Sun, Wenhui Zhou, Shifang Tang

**Affiliations:** 1 School of Foreign Languages, Huazhong University of Science and Technology, Wuhan 430074, China; sunyunmei@hust.edu.cn; 2 Department of Psychology and Special Education, College of Education and Human Services, Texas A&M University-Commerce, Commerce, TX 75428, USA; shifang.tang@tamuc.edu

**Keywords:** adjunct questions, effects on reading comprehension, text types, question types

## Abstract

Answering text-related questions while reading is a questioning strategy which is called adjunct questions or embedded questions, the benefits of which have been established in first-language reading as to enhance comprehension. The present study aims to study the effects different adjunct questions exert on second-language (L2) readers’ comprehension of texts of various types. One hundred and forty-four intermediate-level Chinese EFL learners participated in this study and were divided randomly into six groups. Each group was given either a narrative or an expository text with ‘what or why’ questions or no questions. A brief topic familiarity questionnaire was attached to the end of each text paper. The results showed that inserted adjunct questions improved the readers’ reading comprehension both in expository and narrative texts, but only narrative texts inserted with why questions had significant effects on the L2 reading comprehension. The findings suggested that text types and question types modulate the effects of inserted adjunct questions on the English reading of intermediate learners. Pedagogical implications and suggestions for future studies are provided.

## 1. Introduction

When educated people think about the importance of literacy, most agree that reading ability plays an essential role at school, work, and in society [1]. In fact, reading is considered the basis for later language development [2]. In foreign language classrooms in China, the ability to comprehend academic texts is widely regarded as a crucial competency that college students must develop. However, recent reforms in language education in China have advocated a reduction in classroom language teaching time after the Ministry of Education in China issued a policy on modifying subjects of study in schools and universities. Accordingly, language educators and researchers are now trying hard to find methods to cultivate language learners’ ability to read academic texts independently and efficiently after class, one of which is using adjunct (also called embedded) questions in reading texts. Though no explicit definition of adjunct questions has been given, relevant research performed so far has helped us to provide the following working definition. Adjunct questions refer to content-related or text-related questions inserted into the reading material by language educators requiring readers to answer while reading as these in-text questions help readers recall text information [3,4]. Researchers have found this technique helpful in enhancing reading comprehension in L1 [5].

So far, however, there are limited studies on the effects of adjunct questions on L2 readers [3,4,5,6,7,8,9,10]. Though most researchers have demonstrated a limited benefit of adjunct questions on L2 reading comprehension, a closer examination of relevant literature has revealed that the effects of adjunct questions on reading comprehension were not significant [4] and often varied with different comprehension measurements [10]. Additionally, previous researchers have analyzed the effects of adjunct questions on L2 reading comprehension with either expository texts [3,4,5,10] or scientific texts [9]. In addition, researchers have not reached a consensus on the effect of adjunct questions on L2 reading comprehension. 

Textbooks adopted by universities in China usually include materials on different subjects written in English. Though most of the reading texts are expository in nature, some are narratives. The way to comprehend expository texts differs greatly from that of narrative texts. Narrative texts are basically narrative stories, with clear themes, plots, and key elements that make the text easier to comprehend than expository texts. The present study, therefore, aims at comparing and contrasting the effects that different types of adjunct questions might have on L2 reading comprehension with both expository and narrative texts across different comprehension measurements. The results of this study might offer more experimental evidence for studying the effects of adjunct questions and practical teaching implications for Chinese EFL teachers by providing guidance on training independent and autonomous L2 readers [7].

## 2. Literature Review

Educators and language researchers have been trying for many years to find approaches to cultivating language learners’ ability to read academic texts independently and efficiently. So far, two important methods have been identified. One is to focus on the readers by training them in reading skills or strategies. This has proven to be a slow and complicated process. The other is to focus on the reading texts by increasing the readability of the academic texts (also called textual enhancement) [11]. Both can enhance readers’ independent and autonomous reading ability. In addition, researchers anticipated that textual enhancement could aid the comprehension of reading texts [6]. Inserting questions into reading passages and making analogies are two techniques that are widely used to enhance L1 reading comprehension [7].

### 2.1. Textual Enhancement in L1

Abundant research concerning the textual enhancement of reading comprehension in L1 has been conducted [12,13,14]. They have also identified some essential methods that help improve reading comprehension. Analogies and inserting questions or reading comprehension tasks into the reading texts are considered to be the two most effective techniques. Research using analogies to increase L1 reading comprehension started in the 1980s [15]. Research findings have indicated that it is a successful tool for adults used to obtain new scientific ideas [16], and it proves to be most effective for beginners such as children [15]. 

Research on inserting adjunct questions into reading texts has also been proven effective in the L1 reading process [12,13,14]. The reading comprehension process was described as a process of constructing a mental representation. The mental representation of a text consists of three levels: surface-level representation, text-based verbatim representation, and situational representation [17]. Constructing a situational representation of a text requires readers to find different types of information first. This means readers need to retain the relevant and rule out the irrelevant information. The second layer of the reading process involves making reasonable inferences by clarifying important points not explicitly stated in the text. That means readers will have to fill the information gap they encounter during the reading comprehension process. Readers can use the adjunct questions in the text as an anchor or a starting point. The adjunct questions can help them focus on the critical information, thus activating their prior relevant knowledge about the main topic and constructing a situational representation of a text [6]. 

Previous research on L1 reading has indicated that inserting questions into the reading passages helps to improve readers’ memory of the text, thus enhancing comprehension [5,12,14]. 

### 2.2. Textual Enhancement in L2

Since L2 researchers started investigating the effects of inserted adjunct questions on L2 reading comprehension [5,6,7] they have utilized different methods. Some have tried the analogy method to assist adult L2 comprehension with domain-specific text types [18,19]. When analogies failed to achieve the expected positive effect on L2 reading comprehension, other researchers began to hypothesize the influence of adjunct questions on the L2 reading comprehension because this method has been proven to be beneficial in L1 reading [3,4,5,6,7,8,10,14]. The limited number of studies conducted so far examined and analyzed variables such as learners’ L2 language proficiency level, the embedded question types, methods used to deal with the inserted adjuncts, and the types of assessment tasks for reading comprehension and text types. Variables like topic familiarity are also discussed in some research [4].

#### 2.2.1. Learners’ Language Proficiency

Constructing a situational representation requires the learners to have the ability to remove irrelevant information from the reading texts and to activate the relevant prior knowledge. These abilities are closely related to learners’ language proficiency [14]. It is acknowledged that low-ability readers may have problems distinguishing between relevant and irrelevant information [14]. 

However, research on readers’ language proficiency has revealed that using embedded adjunct questions to enhance readers’ comprehension of both L1 and L2 proved to be more beneficial to low-ability readers than to high-level readers [3,6,9,10,14]. With 97 advanced learners of Spanish, Brantmeier et al. examined the effects of embedded adjunct questions, prior subject knowledge, and L2 Spanish proficiency on the L2 reading comprehension of scientific passages. The results showed that the readers’ L2 proficiency and prior subject knowledge did not enhance the effects of embedded adjunct questions on reading comprehension [9]. A more recent study with intermediate and advanced learners of Chinese found that the adjunct questions only had positive effects on readers of intermediate levels with written recall as a comprehension assessment, but not on advanced L2 learners [10].

#### 2.2.2. Adjunct Question Types

The embedded adjunct questions can be mainly divided into two types: *what* questions and *why* questions. The *what* questions are also referred to as targeted segment (TS) questions, which focus readers on detailed content identified in the text [5,6,7,8,14]. However, the *why* questions, also called elaborative interrogation (EI) questions, attempt to help readers activate their relevant prior knowledge to draw inferences from the reading texts [5,6,8,14]. In a word, *what* questions usually focus on defining concepts, while *why* questions aim at synthesizing and integrating information across different sections of the texts [3].

The effect of *what* questions on improving the memory of the prose has been verified [20,21,22]. *What* questions have also been proven to have positive effects on L2 reading comprehension if questions focus on the same content as the assessment tasks [12]. 

Research on the effect of inserted *why* questions is much more complicated. Findings have shown that the positive effect of *why* questions has been examined in the following aspects: improving the memory of the isolated facts for learners of all ages; helping readers to remember facts in short passages; enhancing the readers’ comprehension of the whole text instead of the fragmental facts in actual prose [13]. Though some researchers have noted that inserting other adjunct questions may achieve better reading effects than *why* questions, this was because the researcher provided a detailed explanation of the text, making the *why* questions unnecessary for the text [5,13].

Besides the studies on the respective effects of *what* and *why* questions on reading comprehension, Liu embedded both types of questions into the exact expository text and identified positive effects on L2 reading comprehension measured by short-answer questions (SAQs), but not multiple-choice questions (MCQs) [4].

Because of the variation in the effects of different types of adjunct questions from prior studies, we need more evidence from research to verify the effects’ differences.

#### 2.2.3. Methods Used to Deal with the Embedded Questions

Research has shown that three methods have been adopted so far to deal with the embedded questions in the reading text. The first method is called “pause-and-consider”, meaning that in the middle of the reading text, readers are asked to pause and consider the embedded questions. The second method is “pause-and-write”, in which in the middle of the reading text, readers are asked to pause and then write down their answers to the embedded questions, either in their native languages or in the target languages. The third method provides no explicit instructions to the readers, leaving blank space for the readers to decide whether to answer the embedded questions. Researchers have used different methods to deal with the embedded questions and have achieved different results.

Brantmeier et al. adopted the third method in their research with a group of British learners of Spanish [6]. Even though they were not required to write down anything, most learners did jot down the answers to the embedded questions in their native language. No significant difference was found in reading assessments between the two groups. They concluded that not asking learners to answer the questions might be the leading reason for the lack of positive effects. Therefore, Brantmeier et al. conducted another study focusing on the effects of different methods on reading comprehension [7]. The findings indicated no significant differences among learners’ reading comprehension, but the group using the pause-and-write method did worst in the written recall task. They attributed this result to the demand of writing down the answers to the questions. Readers might focus too much on the information related to the inserted adjunct questions. This might distract readers’ attention from the process of constructing a clear and coherent mental picture of the text. As a result, other important information that could help readers understand the academic text might be left out. 

Callender et al. adopted the pause-and-write method in their research [5]. Three groups of intermediate-level Spanish learners were asked to answer the embedded questions in the target language. The results indicated that embedded questions did not enhance reading comprehension but only impeded the natural reading process. In fact, the *what* questions showed no effect on learners’ performance in the multiple-choice task, and learners performed significantly worse in the written recall task in both passages tested. The *why* questions had negative effects on the performance of the written recall task. 

The assessment task types and academic text types were considered to be two possible explanations. Therefore, the present study still takes these two variables into consideration.

#### 2.2.4. Assessment Task Types

Four assessment tasks have been used by researchers in the study of textual enhancement. They are multiple-choice questions, written recall, short-answer questions, and sentence completion [3,4,5,6,7,8,10,14,18,23,24]. Research on L2 reading has indicated that the most commonly used assessment tasks were a combination of multiple-choice tasks and written recall [3,5,6,8,10]. The multiple-choice task was chosen because of its efficiency in scoring [6,8], while the written recall task was added to avoid its bias on the final result, especially when Chinese EFL learners were involved [7]. 

Different to multiple-choice tasks, in which both the choices and the answers were predetermined, written recall provided a perfect compensation because a free written recall task enabled readers to select the information they needed to report, and readers were free from any pre-set constraints or limitations [5]. These two assessment tasks were applied to most relevant research to examine L2 learners’ reading comprehension and were proven to complement each other in evaluating L2 learners’ reading performance [3,5,6,7,8,10]. However, there were inconsistent findings concerning the use of these two assessment tasks. Most found that the effects of adjunct questions were not significant when assessed by multiple-choice assessment tasks with advanced readers [6,7,8,9]. When assessed by multiple-choice cloze, the adjunct questions did not promote reading comprehension with intermediate learners of Chinese [10]. The written recall assessment had similar inconsistent findings, with no effects found with Spanish readers [3,5] and promoting effects with Chinese readers [10].

As multiple-choice and written recall tasks are still considered the best combination for assessing readers’ comprehension, this study still utilized these two types of measurements to provide further evidence for research on the effects of adjunct questions with intermediate-level Chinese L2 learners as subjects. The learners were allowed to accomplish the written recall task in either their native language (Chinese) or their target language (English) to ensure that it was a test of reading comprehension rather than a test of writing. 

#### 2.2.5. Text Types

The text type is defined as the structure and organization of the reading materials. 

Hiebert et al. [25] stated that “Extensive first language research (L1) has been conducted on how varied text types affect reading comprehension and some L2 reading research has addressed the same phenomena”. Even though expository and narrative prose have been used most extensively, researchers studying the effects of the embedded questions made the expository text their first choice [4,5,6,7,8,14].

However, different types of text involve different types of processing. According to Callender et al., “expository texts encourage processing individual facts or pieces of information, whereas narratives or proses with an established schema naturally result in relational processing” [5]. 

We thus hypothesize that the *why* questions, which are said to be useful for memorizing isolated facts, will help improve the learners’ reading comprehension of the narrative prose. Narrative text itself induces the processing of relational information, and the reading comprehension of such text types can be enhanced by manipulating textual difficulty. So far, few studies have been conducted on the effects of embedded adjunct questions on reading comprehension of this type, let alone comparing and contrasting the effects between expository texts and narrative texts inserted with different adjunct questions. 

Reviewing prior research on textual enhancement, the present study explores the effects of embedding adjunct questions into narrative and expository texts on the L2 reading comprehension with Chinese intermediate EFL learners using multiple-choice questions and written recall tasks as assessment measures. The following research questions are to be answered in the present study:

(1) What effects do embedded questions have on the intermediate-level Chinese EFL learners’ reading performance?
(2) What effects do the text types and embedded question types have on the intermediate-level Chinese EFL learners’ reading performance?

## 3. Methodology

### 3.1. Participants

About two hundred convenient samples were recruited. After taking a reading comprehension test, only about 153 participants who showed no significant differences in their reading competence joined the formal study (F(5, 138) = 0.827; *p* = 0.533). They were randomly assigned into six different groups. Each group consisted of 25–30 students majoring in chemistry, communication engineering, biology, material engineering, medicine, etc. Their average age was 19.5 years old. Eighty percent were males while 20 percent were females, which was in proportion with the male and female ratio in technology-based universities. All expressed their willingness to participate in the study and signed the consent form in its Chinese version.

These participants had to meet the following three requirements: (1) All had passed College English Test Band 4 (CET 4) but failed in College English Test Band 6 (CET 6). Both tests were large-scale standardized proficiency tests for all Chinese college and university non-English majors. Those who passed CET 4 were rated as intermediate learners of English while those who passed CET 6 were considered to be advanced learners in English proficiency; (2) all had taken the pre-test on reading comprehension to ensure that there were no significant differences in their reading ability; (3) all had completed the experiment tasks required. Nine students failed to meet the above requirements, leaving the number of participants in the final sample to one hundred and forty-four.

### 3.2. Design

This experiment was a 2 × 3 between-group design. Question and text types were independent variables, while participants’ reading comprehension was the dependent variable. We randomly grouped the final 144 participants into one of the six conditions. Three groups dealt with expository text, with 21 participants for *no* question condition, 22 for the *what* questions condition, and 22 for the *why* questions condition. The number of participants who dealt with narrative text was 26, 28, and 25, respectively, in the same condition order as the expository text.

### 3.3. Materials

The materials used for the pre-test were taken from TEM4 (Test for English Majors—level 4), a large-scale standardized test in China that evaluates the language proficiency level of English majors in China. The pre-test consisted of four reading passages with a total of 30 multiple-choice questions on reading comprehension.

The materials used for the experiment consisted of one narrative text and one expository text. The expository text discussed the implicit personality theories in which the attribution theory was expanded in detail. The narrative text described a memorable family trip and family members’ feelings before and after this trip. Both texts were adapted so that they reached similar difficulty levels. The text features of the two English reading materials are presented in Table 1, which were analyzed using Coh-Metrix (3.0) [26].

Table 1 shows the text features of the two passages. Though there were apparent differences in terms of their total word count and L2 readability, we selected the two passages from the same standardized Test for English Majors in China with its reliability and validity being tested by a group of Chinese experts in language testing. Besides, before the formal experiment, we recruited several volunteers at similar English proficiency levels to assess the difficulty level of the two passages. They claimed that these two passages were of similar difficulty for them. Therefore, we adopted these two passages for our study.

Two parallel questions were inserted into each of the two texts for the experimental groups; one was put in the middle of the text, while the other was placed at the end. Both the texts and the questions were presented in English. For example, the inserted *what* questions for text 2 were “What is the parents’ plan to broaden their children’s horizons even though their friends are against it?” and “What did the family see and experience during this trip?” The parallel *why* questions for this text were “Why did the parents stick to the travel plan to Istanbul?” and “Why is this trip so special for the family?” The development of the embedded questions for text 2 was under discussion among a group of experienced language teachers in the university and was aimed at helping the readers recollect individual facts or relational information from what they had read. However, the inserted questions for text 1 were adopted from previous researchers [5]. The two *what* questions inserted in text 1 were “What are implicit assumptions?” and “What are attributions?”. The two parallel *why* questions included “Why do we make implicit assumptions about other personalities?” and “Why do we make attributions?”.

Two assessment tasks were provided for each text: a written recall task and a multiple-choice test. For the written recall task, all participants in the six groups were required to write down as much as they could remember about the text, either in English or Chinese. Allowing participants to use their L1 would rule out the effect of the participants’ writing ability in L2. Both texts had ten multiple-choice questions written in English.

Previous research [4,27] has indicated that background knowledge plays a role in comprehension, so we added a simple questionnaire at the end of the reading texts, testing participants’ familiarity with the topics on a scale of 1–5. 

### 3.4. Procedure

In the eighth week of the fall semester of 2023, a reading test was given to about 200 recruited participants who expressed their willingness to participate in our study. This was convenience sampling. The reading material was taken from College English Test Band 4 (CET4). Based on the results of the test, we excluded about 50 participants whose reading proficiency was either at the top or at the bottom, leaving 150 participants whose reading proficiency was at the same intermediate level. These participants, who volunteered to join our experiment, were randomly assigned into six groups.

These participants all signed the consent form before the experiment started. During their English classroom learning sessions, their teachers gave them instructions on how to finish the reading tasks. After that, each participant received a set of corresponding materials printed on separate sheets. The materials were arranged in the following order: (1) reading passage; (2) topic familiarity questionnaire; (3) written recall task; (4) multiple-choice questions. The two reading texts with six different conditions were distributed randomly among six groups. Participants were told not to read back during the experiment and were given enough time to complete all the tasks. All six groups of participants read one text once. Three groups read text 1, and the other three groups read text 2. Each of the two texts was read under one of the three conditions: no embedded question groups (Groups 1 and 4), embedded *what* question groups (Groups 2 and 5), and embedded *why* question groups (Groups 3 and 6). 

Upon completion, we noted that all participants finished within 45 min. 

### 3.5. Scoring

We used a 100-point grading system. The total score of each participant consisted of two parts: half from the multiple-choice questions and half from written recalls. For multiple-choice questions of the two passages, we divided the 50 points by 10, attributing 5 points to each correct answer learners gave. For example, if a participant gave correct answers to six multiple-choice questions, his total score in this part would be 50/10 × 6 = 30. 

To measure participants’ scores in the written recall task, we calculated the number of pausal units participants could recall from the reading texts. “A pausal unit is a unit or entity that readers feel the need to pause during normally paced oral reading” [23]. Though there were some other scoring rubrics, such as Meyer’s system [25] and Riley and Lee’s “unit of analysis” [28], we still used the pausal unit in the present study because it has been demonstrated to be “more efficient and less time-consuming” and the “most consistently used method to codify written recalls” [5].

Two native English speakers were invited to read the two texts out loud and marked the pausal units in each text. One point was awarded to each pausal unit that was recalled successfully by learners. There were 33 pausal units in text 1 and 29 in text 2. 

Since the two raters’ reliability was very high (r = 0.95), we let each rater score the pausal units separately. After we added the scores of each participant, we divided the total score by 2, thus obtaining the final score for each participant in the written recall task.

## 4. Results

Before reporting the data about the effects of the text types and question types on the L2 reading performance, we conducted a MANOVA on the familiarity ratings for both of the two reading texts by six different conditions. No significant differences were found among the six groups in terms of familiarity (F(5, 129) = 1.590, *p* = 0.167). 

### 4.1. Effects of Embedded Questions on Learners’ Reading Comprehension

The two-way ANOVA analysis was conducted first. Table 2 shows the descriptive statistics.

Table 2 presents the means and standard deviations of the learners’ scores gained in the expository and narrative texts with different assessment tasks under three different question conditions. For the expository text, the differences among the three groups in terms of the learners’ reading comprehension did not reach the significant level (*p* > 0.05). For the narrative text, the learners performed much better in the reading comprehension with the text inserted with *why* questions than with the text with “no” questions or *what* questions. The differences reached a statistically significant level (*p* < 0.01). Generally speaking, the participants obtained higher mean scores on the texts with embedded questions. This suggests that embedded questions may have positive effects on readers’ reading comprehension, especially when inserting *why* questions into narrative texts.

To further understand the effects, we conducted an ANOVA analysis to test the effects of the question types and text types on the learners’ reading performance. Table 3 provides the details. 

Table 3 shows that the main effect of both the text type (F(1, 138) = 8.80, *p* = 0.00) and question type (F(2, 138) = 3.81, *p* = 0.03) reached significant levels. The interaction effect of the expository text and narrative text with the different types of questions was also significant (F(2, 138) = 5.78, *p* = 0.00). This indicates that the text types and question types may have interacted together and significantly affected the participants’ reading performance. Therefore, a simple effect analysis was required to obtain further interpretation.

### 4.2. Effects of Question Types and Text Types on Learners’ Reading Comprehension

#### 4.2.1. Effects of Question Types on Learners’ Reading Comprehension

We conducted a simple interaction effect analysis. Table 4 presents the specific effects the question types imposed on the learners’ reading comprehension.

Table 4 shows that significant differences exist only between inserting the *no* questions and *why* questions into the reading texts (*p* = 0.03) when the text type is not taken as a variable. There was no significant difference in the reading comprehension between the texts with *no* embedded questions and the texts with *what* questions (*p* = 0.34), and between the embedded *what* and *why* questions (*p* = 0.48). 

#### 4.2.2. Effects of Text Types on Learners’ Reading Comprehension

Table 5 shows the results of the simple effect analysis of the text type on the learners’ reading comprehension. 

Table 5 shows that when the adjunct questions were inserted into the expository text, no significant difference was found in the learners’ reading comprehension (F(2, 138) = 2.46, *p* = 0.09). However, when the narrative text was inserted with different questions, a significant difference was found (F(2, 138) = 6.55, *p* = 0.00). Table 6 provides detailed information. 

Table 6 shows that in dealing with the narrative text, the learners with the embedded *why* questions obtained much higher mean scores (M = 45.85) in the reading comprehension test than the learners with *no* questions (M = 36.81) or with *what* questions (M = 37.06). However, when dealing with the expository text, there were few differences among the three groups.

#### 4.2.3. Interactive Effects of Question Types and Text Types on Learners’ Reading Comprehension

Table 7 shows the interactive effects between the text types and question types.

Table 7 indicates that variable *text type* had no effect on the learners’ reading comprehension when the texts were embedded with *no* questions (F(1, 138) = 1.88, *p* = 0.17) or with *what* questions (F(1, 138) = 0.36, *p* = 0.55). Positive effects on the learners’ reading comprehension were only found in the texts inserted with *why* questions (F(1, 138) = 17.21, *p* = 0.00). This might imply that inserting *why* questions into different types of reading helps enhance the L2 reading comprehension. However, Table 5 showed that inserting questions into expository texts did not have any positive effects on the learners’ reading comprehension (*p* > 0.05). Thus, we could conclude that the positive effects only occur when inserting *why* questions into narrative texts. 

## 5. Discussion

The present study found that adjunct questions had no facilitating effects on the intermediate Chinese EFL readers’ reading performance with the expository texts but strong positive effects with the narrative texts. We also found that the positive effect of the adjunct questions only emerged for the written recall measurement but not for the multiple-choice tasks when we inserted *why* questions into narrative texts with written recall measurements. Significant interactive effects were also found between the embedded question types and text types. 

The findings of no significant effects on the Chinese EFL learners’ reading performance with the expository text measured by multiple-choice tasks were consistent with prior L2 research [6,7,8,9,10]. One possible explanation is that the processing of the text produced by adjunct questions is redundant with the spontaneous processing of the readers, especially for more proficient readers. The context of expository texts may offer relevant explanations for the adjunct questions, thus making the questions ineffective because the answers were already embedded in such texts.

The results of inserting *why* questions into the narrative text eliciting better-written recalls for the Chinese EFL learners did not align with some previous Spanish studies [3,5] but were in agreement with a previous study in a Chinese setting [10]. One plausible explanation is related to the cognitive skills involved. The cognitive skills required to complete the written recall task and cognitive process triggered to answer *why* questions are similar in that “both involve recognizing, recalling and reorganizing textual information in a coherent manner” [29]. Both written recalls and *why* questions are open-ended questions, the completion of which requires information recollection [30].

This finding also refuted the statement that adjunct questions limited the L2 readers’ processing of the text. The embedded *why* questions helped activate the L2 readers’ prior knowledge with a scaffold to create a cognitive map of the text [5,6,8,14]. The Chinese learners’ language learning habits, as well as their language proficiency level, might also be attributed to the results. Chinese EFL learners today do not think that language learning only involves simple repetition. Rather, they believe that memorization, understanding, practicing, and reviewing all contribute to better understanding and learning a second language. Accordingly, when confronted with a reading passage, Chinese EFL learners tend to memorize as much as they can. These inserted *why* questions act like anchors to recollect and reconsider what they have read and relate it to their prior knowledge. As a result, they tend to be more familiar with the content after this recollecting process and are likely to accomplish the written recall assessment tasks more efficiently. 

Apart from the explanation of the Chinese learners’ language learning habits, the positive effects of the embedded *why* questions could be due to their language proficiency levels. As has been hypothesized in prior research [10,14], the effects of the adjunct questions might vary with the learners’ comprehension abilities. For low-level readers, their ability to activate prior relevant knowledge and construct mental representations for passages is relatively limited. These inserted adjuncts, especially the *why* questions, require readers to understand the text and use their prior knowledge to answer. This would bring extra burden to the low-level learners and further exacerbate the difficulties they face in completing the construction of a cognitive map of the text. For high-level readers, the negative effects of the inserted questions have been proven repeatedly in previous research [6,14]. The advanced learners were considered naturally competent to construct a corresponding representation of the material directed by adjunct questions which would distract their correct understanding of the text. 

Learners at the intermediate proficiency level already have a certain ability to construct cognitive representations for the text, but have not yet reached the same proficiency level as the high-level construct builders. This makes the adjunct questions work as an anchor. Questions are used as a standing point to allow the readers to strengthen their memory, understand the passage, and complement the learners’ reading ability. That is why the embedded *why* questions positively affected the intermediate-level learners’ reading comprehension.

The interactive effects found between the text types and question types might be because different text types provide different ways of processing information. According to the Material Appropriate Processing (MAP) framework, a good recall of a text depends largely on the ability to encode both individual-item and relational information. Each text type is supposed to have its information processed in its own way. The difficulty of manipulation (here, in the present study, it refers to the adjunct questions) is considered to help stimulate new ways of processing texts. 

The nature of the narrative text and the inserted *why* questions in our study helped enhance the learners’ reading comprehension in the written recall because narrative text encourages processing relational information, while the inserted *why* questions help to stimulate individual-item information [5,31].

Different to the embedded *why* questions, the *what* questions encouraged the readers to center around the surface information or text-based information [7]. If the readers were in remedial reading programs and in need of identifying specific parts of the text, this might be quite useful as they provide additional support to readers [5,6,7,8,14,31]. In the present study, the reading tasks required the readers to process not only the surface information but also deep structure information. That could explain why inserted *what* questions could improve learners’ reading comprehension to a certain extent, but the difference did not reach a significant level.

## 6. Conclusions and Implications

The present research found that adjunct questions have positive effects on Chinese intermediate EFL learners with narrative texts inserted with *why* questions. The interactive effects of question types and text types have also been identified. The inserted *why* questions in narrative texts are related to item-specific information focusing on surface or text-based level information while *what* questions mainly focus on relational information.

Language educators should realize the importance of reading competence and the effects of reading on students’ educational achievement. This is especially true in the current and future Chinese L2 context, with classroom teaching time largely reduced. Therefore, for educators, it is important that they train students to become independent and autonomous readers. Textbook writers, who aim to teach learners effective reading skills and to improve learners’ independent reading competence, should include different types of reading materials and offer training in other reading techniques to L2 readers. One limitation of this study is that the participants’ proficiency level was rated using a localized test, CET 4, instead of an internationally recognized test such as TOEFL or IELTS. Another limitation is that the two texts we selected for our study differ not only in text type, but also in themes, subject, word count, and readability. These differences might affect the results. Future studies could center around investigating other types of adjunct responses, other test types, the effect of topic familiarity, and using other methods (such as think-aloud or eye-tracking techniques) to explore learners’ cognitive processes. In addition, when selecting materials, issues such as the text type, readability, theme, and subject should all be considered.

## Figures and Tables

**Table 1:** Text features of the two passages.

Text	Word Count	No. of Sentences	Type–Token Ratio	L2 Readability
T1	546	23	0.52	10.15
T2	703	27	0.53	14.07

Note: T1: Expository text; T2: Narrative text; Type–token ratio: The number of unique words divided by the number of tokens of the words to indicate lexical diversity. Readability: Calculated based on content word overlap, sentence syntax similarity, and lexical frequency to indicate L2 text difficulty.

**Table 2:** Descriptive statistics by text and group conditions.

T	Test Type	Control			Treatment 1			Treatment 2		
		Mean	SD	N	Mean	SD	N	Mean	S.D	N
T1	M.C	5.46	1.64	21	6.7	1.69	22	6.75	2.07	22
	W.R	3.13	2.54		3.91	2.31		2.00	1.22	
	Total	32.19	11.27		38.86	9.96		34.15	8.99	
T2	M.C	4.69	1.59	26	4.07	1.28		5.92	1.22	25
	W.R	6.23	3.10		7.86	2.98	28	7.52	3.18	
	Total	36.81	11.96		37.06	7.73		45.85	8.06	
Total		34.75	11.76	47	37.85	8.73	50	40.38	10.28	47

Note: T1: expository text; T2: narrative text; Control: reading with no embedded questions; Treatment 1: reading with embedded *what* questions; Treatment 2: reading with embedded *why* questions; N: number of participants.

**Table 3:** Tests of effects between text types and question types.

Source	Type III Sum of Squares	df	Mean Square	F	*p*
Text	833.68	1	833.68	8.80	0.00
Question	721.56	2	360.78	3.81	0.03
Text × Question	1094.54	2	547.27	5.78	0.00
Error	13,071.87	138	94.72		
Total	219,963.41	144			

**Table 4:** Effects of question types on reading comprehension.

(I) Question Type	(J) Question Type	Mean Difference (I–J)	Std. Error	*p*
Type 1	Q2	−3.10	2.09	0.34
Type 2	Q3	−5.63	2.12	0.03
Type 3	Q3	−2.53	2.09	0.48

Note: Scheffe method used to make the comparison; Type 1: text with no questions; Type 2: text embedded with *what* questions; Type 3: text embedded with *why* questions.

**Table 5:** Simple effects of text types on reading comprehension.

Source of Variation	SS	df	MS	F	*p*
Within cell	13,071.87	138	94.72		
Questions (T1)	493.02	2	246.51	2.46	0.09
Questions (T2)	1310.21	2	655.1	6.55	0.00

Note: T1: expository text; T2: narrative text.

**Table 6:** Interaction effect between text types and question types.

Text	Question Type	Mean	Std. Error	95% Confidence Interval	
				Lower Bound	Upper Bound
T1	Q1	32.19	2.12	27.99	36.39
	Q2	38.86	2.08	34.76	42.96
	Q3	34.15	2.08	30.05	38.26
T2	Q1	36.81	1.91	33.04	40.59
	Q2	37.06	1.84	33.42	40.69
	Q3	45.85	1.95	42.01	49.70

Note: T1: expository text; T2: narrative text; Q1: text with no embedded questions; Q2: text embedded with *what* questions; Q3: text embedded with *why* questions.

**Table 7:** Interactive effects of text types and question types.

Source of Variation	SS	DF	MS	F	*p*
Within cells	13,071.87	138.00	94.72		
Text within (Q1)	185.41	1.00	185.41	1.88	0.17
Text within (Q2)	35.72	1.00	35.72	0.36	0.55
Text within (Q3)	1695.15	1.00	1695.15	17.21	0.00

Note: Q1: text with no embedded questions; Q2: text embedded with *what* questions; Q3: text embedded with *why* questions.

## Data Availability

The data supporting this study’s outcomes are accessible from the corresponding author. Restrictions apply to the availability of these data, which were utilized with a license for this study.
